# Selective Ablation of Ctip2/Bcl11b in Epidermal Keratinocytes Triggers Atopic Dermatitis-Like Skin Inflammatory Responses in Adult Mice

**DOI:** 10.1371/journal.pone.0051262

**Published:** 2012-12-20

**Authors:** Zhixing Wang, Ling-juan Zhang, Gunjan Guha, Shan Li, Kateryna Kyrylkova, Chrissa Kioussi, Mark Leid, Gitali Ganguli-Indra, Arup K. Indra

**Affiliations:** 1 Department of Pharmaceutical Sciences, College of Pharmacy, Oregon State University, Corvallis, Oregon, United States of America; 2 Molecular Cell Biology Program, Oregon State University, Corvallis, Oregon, United States of America; 3 Environmental Health Science Center, Oregon State University, Corvallis, Oregon, United States of America; 4 Department of Dermatology, Oregon Health and Science University, Portland, Oregon, United States of America; CNRS-University of Toulouse, France

## Abstract

**Background:**

Ctip2 is crucial for epidermal homeostasis and protective barrier formation in developing mouse embryos. Selective ablation of Ctip2 in epidermis leads to increased transepidermal water loss (TEWL), impaired epidermal proliferation, terminal differentiation, as well as altered lipid composition during development. However, little is known about the role of Ctip2 in skin homeostasis in adult mice.

**Methodology/Principal Findings:**

To study the role of Ctip2 in adult skin homeostasis, we utilized Ctip2^ep−/−^ mouse model in which Ctip2 is selectively deleted in epidermal keratinocytes. Measurement of TEWL, followed by histological, immunohistochemical, and RT-qPCR analyses revealed an important role of Ctip2 in barrier maintenance and in regulating adult skin homeostasis. We demonstrated that keratinocytic ablation of Ctip2 leads to atopic dermatitis (AD)-like skin inflammation, characterized by alopecia, pruritus and scaling, as well as extensive infiltration of immune cells including T lymphocytes, mast cells, and eosinophils. We observed increased expression of T-helper 2 (Th2)-type cytokines and chemokines in the mutant skin, as well as systemic immune responses that share similarity with human AD patients. Furthermore, we discovered that thymic stromal lymphopoietin (TSLP) expression was significantly upregulated in the mutant epidermis as early as postnatal day 1 and ChIP assay revealed that TSLP is likely a direct transcriptional target of Ctip2 in epidermal keratinocytes.

**Conclusions/Significance:**

Our data demonstrated a cell-autonomous role of Ctip2 in barrier maintenance and epidermal homeostasis in adult mice skin. We discovered a crucial non-cell autonomous role of keratinocytic Ctip2 in suppressing skin inflammatory responses by regulating the expression of Th2-type cytokines. It is likely that the epidermal hyperproliferation in the Ctip2-lacking epidermis may be secondary to the compensatory response of the adult epidermis that is defective in barrier functions. Our results establish an initiating role of epidermal TSLP in AD pathogenesis via a novel repressive regulatory mechanism enforced by Ctip2.

## Introduction

Mammalian skin forms the first defense barrier in the body for protecting against physical injuries, ultraviolet radiation, bacterial infections as well as excessive loss of water [1]. The most abundant cell type in epidermis is keratinocytes, which forms four layers: basal, spinous, granular and stratum corneum [1], [2]. Atopic dermatitis (AD) is a chronic, inflammatory disease of the skin that starts at early childhood. AD patients are genetically predisposes to the disease, which has a prevalence of 10%–20% in children and 1%–3% in adults [3], [4]. Clinical features of AD include skin xerosis, pruritus and eczematoid skin lesions [4]. AD is characterized by both skin barrier deficiencies and immunological responses [5]. In AD, a defective skin barrier is thought to permit the penetration of allergens and induces the interactions of the allergens with immune cells, promoting the subsequent release of pro-inflammatory cytokines and chemokines and elevation of IgE level [6].

The molecular pathways involved in AD pathogenesis remain unclear. There have been many reports on the connections between atopic dermatitis and Th2 inflammatory pathways [4], [7], [8]. Pro-Th2 cytokines, such as IL-4, IL-13, IL-5, and IL10, are elevated in AD patients [7]. One possible candidate that has been linked to the initiation of inflammatory responses in AD and other allergic diseases is thymic stromal lymphopoietin (TSLP), a member of the hematopoietic cytokine family. It is expressed at a low level primarily by epithelial cells, including keratinocytes, upregulated in acute and chronic AD lesions and responsible for activating cutaneous DCs, endowing them with the capacity to polarize CD4^+^ T cells toward a Th2 cell allergic response [9]–[12]. TSLP signals through a heterodimeric receptor formed by TSLP receptor (TSLPR) as well as IL-7 receptor alpha (IL7Rα) [9]. Mice that are deficient of nuclear receptors (NRs) retinoid X receptor α (RXRα), vitamin D receptor (VDR) or Notch1 in epidermal keratinocytes, exhibit epidermal permeability barrier (EPB) defects, express elevated levels of TSLP in skin, and subsequently develop AD-like phenotypes [10], [12]–[14].

Chicken ovalbumin upstream promoter transcription factor (COUP-TF) interacting protein 2 (Ctip2), also known as Bcl11b, is a C_2_H_2_ zinc finger protein that is crucial in the development of central nervous and immune system [15]–[18]. In T cells and neuroblastoma cells, Ctip2 appears to function predominantly as a transcription repressor. Ctip2 interacts indirectly with histone deacetylases (HDACs) within the context of the Nucleosome Remodeling and Deacetylation (NuRD) or SIRT1 complexes [16], [19], [20]. Ctip2 also acts as a transcriptional activator in a promoter-dependent manner in stimulated thymocytes [21], [22]. Ctip2 is also expressed in human and mouse adult epidermis [23], [24]. Studies with mice harboring germline deletion and epidermal specific ablation of Ctip2 revealed its critical role(s) in epidermal proliferation and terminal differentiation, as well as barrier formation during mouse embryonic development in both cell autonomous and non-cell autonomous ways [25]. Ctip2 was shown to be an important regulator of epidermal homeostasis and barrier formation by interacting with the promoter regions of many genes involved in epidermal development such as EGFR and Notch1 and in skin lipid metabolism such as eLox3, Gba2 and Lass2 ([26], Wang et al., 2012, in press). Since Ctip2-null mice die at birth, the function(s) of keratinocytic Ctip2 in adult mice EPB maintenance as well as in epidermal homeostasis is unknown.

Here we report a novel role of Ctip2 in maintenance of adult epidermal homeostasis and in skin inflammation by selective Cre-recombinase mediated ablation of Ctip2 gene in epidermal keratinocytes of mice skin [27], [28]. We show that ablation of Ctip2 in mice epidermal keratinocytes during development results in impaired EPB maintenance, increased epidermal hyperplasia and a severe form of AD-like skin inflammation of adult skin that becomes disseminated with progression of the disease, all of which are very similar to AD in humans. Our present data indicates that keratinocytic Ctip2 plays a crucial role in triggering skin inflammatory responses by regulating the expression of genes encoding Th2-type cytokines in adult mouse skin. These results presented herein establish an initiating role of epidermal TSLP in AD pathogenesis and demonstrate a key anti-inflammatory role of Ctip2, which strongly represses TSLP expression in wild-type skin.

## Results

### Selective ablation of Ctip2 in epidermal keratinocytes leads to altered epidermal proliferation, differentiation and spontaneous dermatitis in adult skin

**Figure 1:**
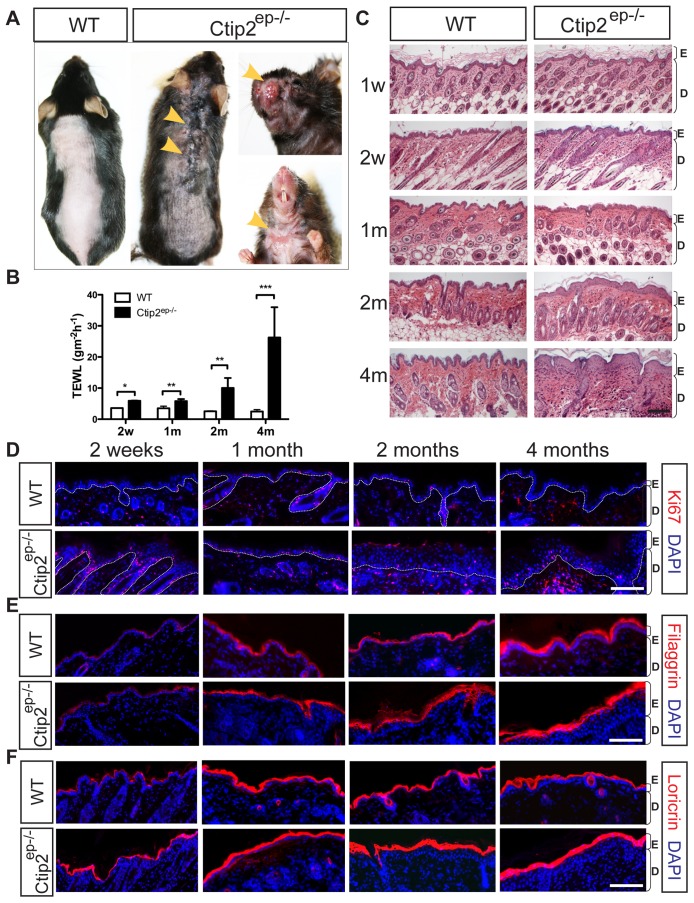
Ctip2^ep−/−^ mice develop a chronic skin lesions and enhanced epidermal proliferation and differentiation. (**A**) Gross morphology of 4 months old wildtype (WT) and Ctip2^ep−/−^ mice. The yellow arrowheads indicate lesion and alopecia of Ctip2^ep−/−^ mice in the back, face and neck, to be compared with the normal appearance in a wildtype mouse. (**B**) Measurement of trans-epidermal water loss (TEWL) from dorsal skin of wildtype and Ctip2^ep−/−^ mice at different time points. Statistical analyses were performed by student's unpaired t-test using GraphPad Prism software; ** P<0.01, *** P<0.001. (**C**) Hemotoxylin & Eosin stained 5 µm thick paraffin sections from dorsal skin of WT and Ctip2^ep−/−^ mice at 1 week (1W), 2 weeks (2w), 1month (1 m), 2 months (2 m) and 4 months (4 m). Immunohistochemical staining of dorsal skin biopsies from WT and Ctip2^ep−/−^ mice was performed with antibodies directed against (**D**) Ki67, (**E**) Filaggrin and (**F**) Loricrin (all in red). All sections were counterstained with DAPI (blue). Scale bar: 100 µm. Epidermis (E) and dermis (D) are indicated.

The function of Ctip2 in adult mice skin homeostasis was characterized using Ctip2^ep−/−^ mice that selectively lacked Ctip2 in epidermal keratinocytes by Cre-recombinase mediated deletion of Ctip2 gene in keratinocytes using K14-Cre deleter mice [27], [28]. At approximately 8–10 weeks after birth, 67% Ctip2^ep−/−^ mice (n = 18) developed dry and scaly skin and 17% mutant mice also developed spontaneous lesions on the dorsal skin at the same age, which were absent in wildtype (WT) mice (Fig. 1A). The severity of all of these abnormalities worsened with age (Figure 1A). By 4 months of age, almost all the mutants (89%, n = 18) developed spontaneous dermatitis that occurred predominantly on the dorsal skin, in the neck region, and on the face (Figure 1A). Furthermore, progressive alopecia was evident in most of the 4 month-old Ctip2^ep−/−^ mice (∼85%) (Figure 1A). We hypothesized that the skin lesions observed in the mutant mice could be due to impaired function of the skin barrier. To test this hypothesis, trans-epidermal water loss (TEWL) was measured in wildtype and Ctip2^ep−/−^ mice skin. Interestingly, the mutant mice showed increased TEWL as early as 2 weeks of age, and the values were ∼10-fold higher than wildtype mice at 4 months of age (Figure 1B; note that 2 M and 4 M TEWL were measured on lesional skin sites). At 4 M, TEWL of nonlesional mutant skin was a third of the TEWL of lesional skin, but it was still 3.5-fold higher than WT controls (Figure S1B). Histological analysis of hematoxylin- and eosin- (H&E) stained skin sections revealed significant epidermal hyperplasia in Ctip2^ep−/−^ mice as early as 2 weeks of age (Figures 1C and S1A).

In order to determine if increased epidermal hyperplasia could be due to altered keratinocyte proliferation, immunohistochemical (IHC) analyses for proliferation marker Ki67 and keratin 14 (K14) were performed on control and mutant skin (Figures 1D and S1C–D). The percentage of Ki67-positive cells was significantly higher in Ctip2^ep−/−^ epidermis compared to the wildtype littermates at all time-points (Figure 1D, S1C). Similarly, the expression level of K14 in the mutant epidermis was prominently increased in Ctip2^ep−/−^ mice at 2 and 4 months of age (Figure S1D). Expression levels of the early differentiation marker K10 were not significantly different between wildtype and Ctip2^ep−/−^ mice (Figure S1E). Expression of terminal differentiation marker filaggrin was reduced in 1-week-old mutant epidermis (Figure S1F); however, the decrease of its expression was transient, as filaggrin expression became similar between WT and in Ctip2^ep−/−^ mice at later timepoints from 2 weeks to 4 months (Figure 1E, S1F–G). The expression of loricrin was similar between wildtype and Ctip2^ep−/−^ mice at all timepoints (Figure 1F). These results indicate that selective, somatic ablation of Ctip2 in epidermal keratinocytes leads to increased epidermal proliferation and unaltered terminal differentiation during adulthood. Altogether, results suggest that adult Ctip2^ep−/−^ mice exhibited impaired barrier functions and developed spontaneous dermatitis that further aggravated with age.

### Increased infiltration of inflammatory cells in adult Ctip2^ep−/−^ mice skin

**Figure 2:**
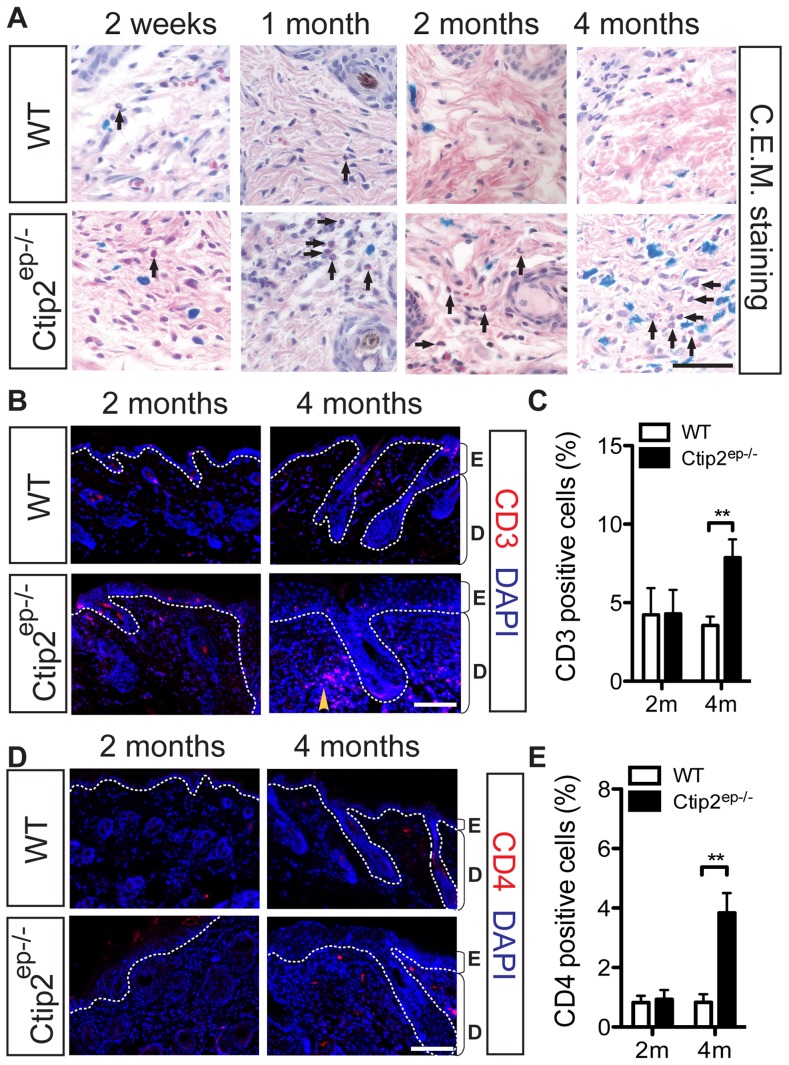
Characterization of inflammatory cell infiltrates in dorsal skin of WT and in Ctip2^ep−/−^ adult mice. (**A**) Combined eosinophil and mast cell (C.E.M) staining for eosinophils (pink) and mast cells (blue). Black arrows point to eosinophils. Scale bar: 50 µm. (**B**) Immunohistochemical staining of dorsal skin biopsies from WT and Ctip2^ep−/−^ mice were performed with specific antibodies against CD3 (red). Yellow arrowhead indicates dermal infiltrates of CD3+ cells. Scale bar: 100 µm. (**C**) Percent CD3+ T cells at 2 m and 4 m. (**D**) Immunostaining of CD4+ T cells (red) in WT and mutant mice. Scale bar: 100 µm. (**E**) Percent CD4+ T cells at 2 m and 4 m. All sections were counterstained with DAPI (blue). Statistical analyses were performed by student's unpaired t-test using GraphPad Prism software; ** P<0.005.

**Figure 3:**
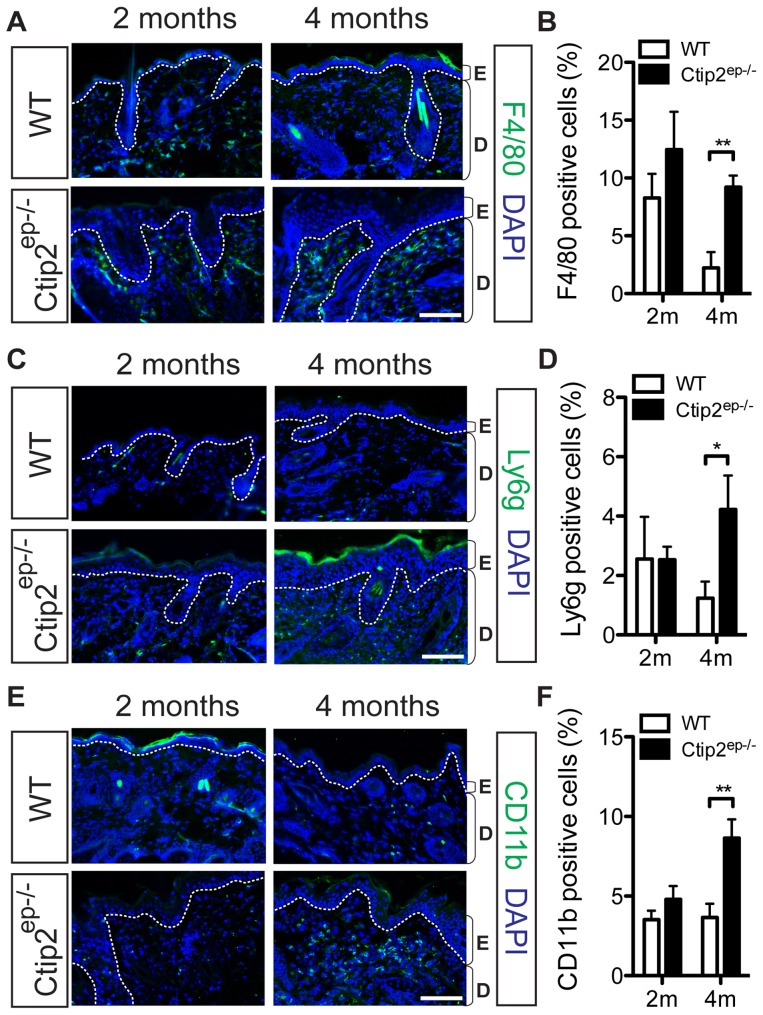
Increased inflammatory cell infiltrate in Ctip2^ep−/−^ adult mice. (**A**) Immunohistochemical staining of dorsal skin biopsies from WT and Ctip2^ep−/−^ mice were performed with specific antibodies against F4/80 (green) to detect macrophage/dendritic cells. (**B**) Percent F4/80 positive cells at 2 m and 4 m. (**C**) Immunostaining of neutrophils (green) using anti-Ly6g antibody in WT and mutant mice skin. (**D**) Percent Ly6g positive cells at 2 m and 4 m. (**E**) Immunostaining of monocytes/macrophages (green) with anti-CD11b antibody in WT and mutant mice skin. (**F**) Percent CD11b positive cells at 2 m and 4 m. All sections were counterstained with DAPI (blue). Scale bar (A, C, and E): 100 µm. Statistical analyses were performed by student's unpaired t-test using GraphPad Prism software; * P<0.05, ** P<0.005.

Dermal infiltrates were notable in Ctip2^ep−/−^ mice starting around 2 months of age, as revealed by histological analyses (Figure 1C). Combined eosinophil and mast cell (C.E.M.) staining revealed increased eosinophils in the dermis of Ctip2^ep−/−^ mutant skin around 1 month of age, while mast cells were most numerous in the mutant dermis around 2 months of age (Figures 2A and S2A). Besides the early induction of eosinophil and mast cells, IHC staining for T lymphocytes revealed a two-fold increase in CD3^+^ T cells mainly at later timepoint in the skin of 4 month-old Ctip2^ep−/−^ mice compared to the wild-type skin (Figures 2B and C). A similar late increase in the number of CD4^+^ T cells was observed in 4 month-old mutant skin compared to the wildtype skin (Figure 2D–E). The number of F4/80+ macrophages in the dermis was significantly higher in Ctip2^ep−/−^ mice skin at 4 months of age (Figure 3A–B). IHC staining using anti-Ly6g antibody showed significantly increased number of neutrophils in the dermis of 4 months old mutant skin (Figure 3C and D). Similarly, staining with anti-CD11b antibodies revealed significantly increased number of infiltrating monocytes, in the 4 months old mutant dermis compared to the wild-type dermis (Figure 3E–F). In addition, CD45^+^ leukocyte infiltration was observed in one month-old Ctip2^ep−/−^ mice skin, but this resolved by 4 months of age (Figure S2B–C). Expression of CD11c positive antigen for dendritic cells was similar between wildtype and Ctip2^ep−/−^ mice skin (Figure S2D). Taken together, these results demonstrate an increased inflammatory response in Ctip2^ep−/−^ skin that progressively increased with time.

### Preferential induction of Th2-type cytokines and chemokines in adult Ctip2^ep−/−^ skin

**Figure 4:**
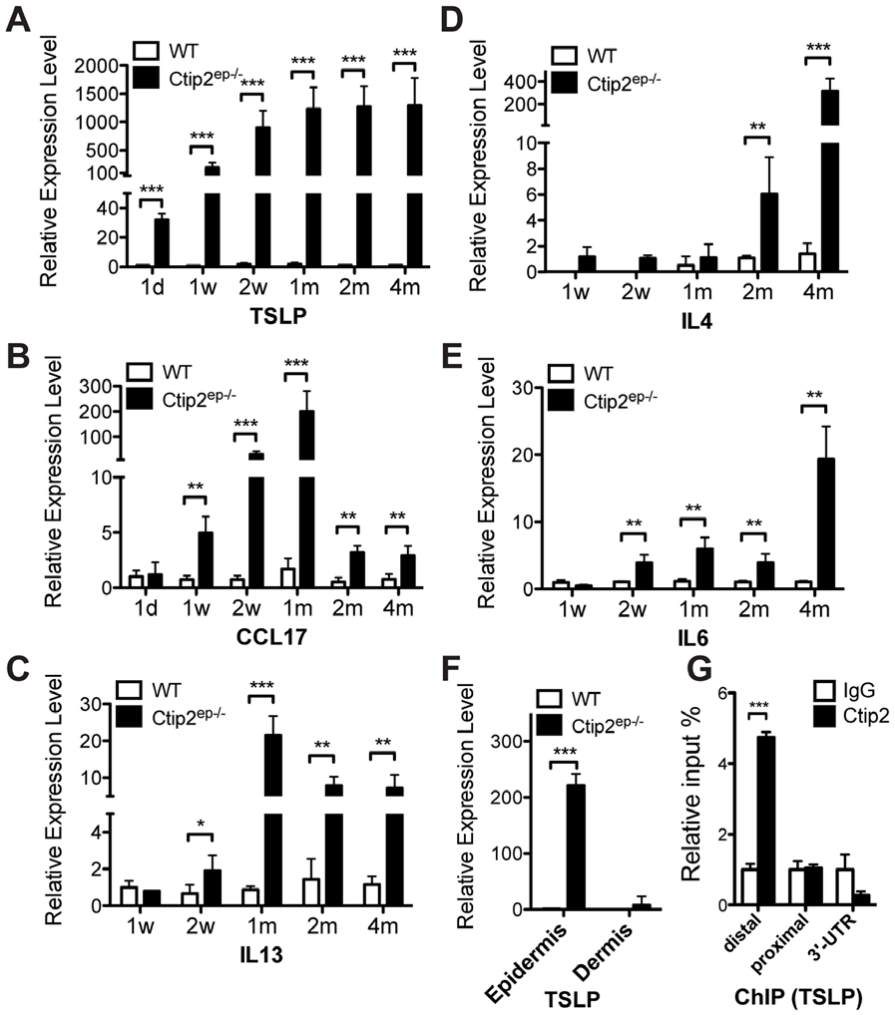
Th2-dependent cytokine and chemokine expression levels in WT and Ctip2^ep−/−^ skin. (**A–F**) Quantitative RT-PCR (RT-qPCR) analyses of cytokines and chemokines in the dorsal skin of 1day, 1 week, 2 weeks, 1month, 2 months and 4 months old WT and mutant mice using specific primers as indicated in Table S1. Relative mRNA expression levels of (**A**) TSLP, (**B**) CCL17, (**C**) IL13, (**D**) IL4, and (**E**) IL6 in Ctip2^ep−/−^ skin was compared to wildtype (WT) skin (set as 1.0). (**F**) RT-qPCR analyses of TSLP mRNA levels in separated epidermis and dermis from tail skin. All values represent relative transcript level after normalization with HPRT transcripts. (**G**) Chromatin immunoprecipitation (ChIP) assay was performed on freshly isolated neonatal mouse skin keratinocytes using anti-Ctip2 antibody and results were analyzed by qPCR using primers indicated in Table S1. Rat IgG was used as a control. Ctip2 was recruited to the distal promoter regions of TSLP. Statistical analyses were performed by student's unpaired t-test using GraphPad Prism software; * P<0.05, ** P<0.01, *** P<0.001.

Upregulated expression of cytokines and chemokines play a crucial role in inflammatory skin diseases [7], [29]. Differential expression profiles of cytokines and/or chemokines orchestrate and determine the type and outcome of inflammatory responses in mouse and human skin. We next determined if expression of pro-inflammatory cytokines/chemokines were dysregulated in mutant skin. RT-qPCR analyses revealed a preferential induction of Th2-type cytokines, including TSLP, CCL17, IL13, IL4, IL6, and IL10 in mutant skin (Figures 4A–E and S3A). In contrast, expression of Th1-type (IL1α, IL2, IL12a/b; Figure S3B–E) was reduced and that of Th17-type (IL17a, IL18 and IL23; Figure S3F–H) cytokines was not induced in mutant skin.

Time course analyses also revealed several distinct induction patterns of cytokines and chemokines in the mutant skin. Chemokine CCL17 is one of known downstream genes whose expression can be stimulated by TSLP [30]. CCL17 and IL13 transcripts were significantly induced in the mutant skin at around 1 week and 2 weeks after birth, respectively. Induction of these two genes was transient, peaking at 1 month, followed by a decrease of expression during later time points (2–4 months; Figure 4B and C). In contrast to these early and transient inductions, we observed a strong, late induction of cytokines IL4 and IL6 and chemokines CCL3 and CXCL2 in the mutant skin (Figure 4D–E, S3L–M). We also observed a late onset of induction of Th1-type cytokines, including TNFα and IFNγ, at 4 months of age (Figure S3N–O).

TSLP, a master initiator of Th2 inflammatory responses, was induced ∼30-fold at postnatal day 1 (P1), and we observed a ∼200-fold induction at P7 compared to control skin [31]. Expression of TSLP remained significantly high at all later time points (∼1000-fold induction between 1 month to 4 months after birth; Figure 4A). Interestingly, the high level of TSLP expression was observed predominantly in the mutant epidermis, whereas TSLP transcripts were barely detectable in the dermal layer of the mutant skin (Figure 4F). Immunohistochemistry analyses revealed that TSLP protein was barely detectable and restricted to differentiating layers of epidermis in WT skin, whereas TSLP protein was strongly upregulated throughout the epidermis including basal proliferative cells and differentiating keratinocytes in the mutant skin (Figure S4A). These data provide strong evidence that TSLP may be an initial triggering cytokine that is induced in the absence of Ctip2 in the epidermis. Altogether, these results suggest that deletion of Ctip2 in the epidermis leads to an increased inflammatory response, which is characterized by a Th2-type cytokine/chemokine profile.

### TSLP is a direct target of Ctip2 in epidermal keratinocytes

Recent studies have revealed that ablation of RXRα and RXRβ in adult murine keratinocytes leads to an AD-like phenotype triggered by induction of TSLP [13].Similarly, TSLP is also elevated in Notch signaling-deficient keratinocytes (deficient in either Notch1/2 or recombining binding protein suppressor of hairless (RBP-j) [12], [13], [32]. Thus, it is possible that Ctip2 regulates TSLP expression via the nuclear receptor or Notch signaling pathways in epidermis. To test that, we determined the expression of key players in both the Notch and RXR signaling pathways, including Notch1, Notch2, RBP-j, RXRα and RXRβ. Expression of the corresponding transcripts was not affected by loss of Ctip2 in the epidermis at both one and four months after birth (Figure S5A–D, data not shown). Similarly, epidermal protein levels of Notch1 and RXRα were comparable between wildtype and mutants at all time-points, suggesting an alternative mechanism of Ctip2-mediated regulation of TSLP expression in the adult murine skin (Figure S5E–F).

In order to determine if Ctip2 directly regulates TSLP expression in murine keratinocytes, we conducted chromatin immunoprecipitation (ChIP) assays on neonatal mouse epidermal extracts. Multiple primer sets were designed from the proximal (−200 bp) and distal (−1 kb) promoter regions relative to the transcriptional start site of TSLP to determine the possible interaction of Ctip2 with TSLP. We observed that Ctip2 interacted with the distal but not the proximal region of TSLP promoter, suggesting Ctip2 may directly repress TSLP transcription in mouse keratinocytes (Figure 4G). Hence, ablation of Ctip2 in epidermal keratinocytes could lead to upregulation of TSLP expression due a derepression mechanism.

### Systemic inflammatory responses in older Ctip2^ep−/−^ mice

**Figure 5:**
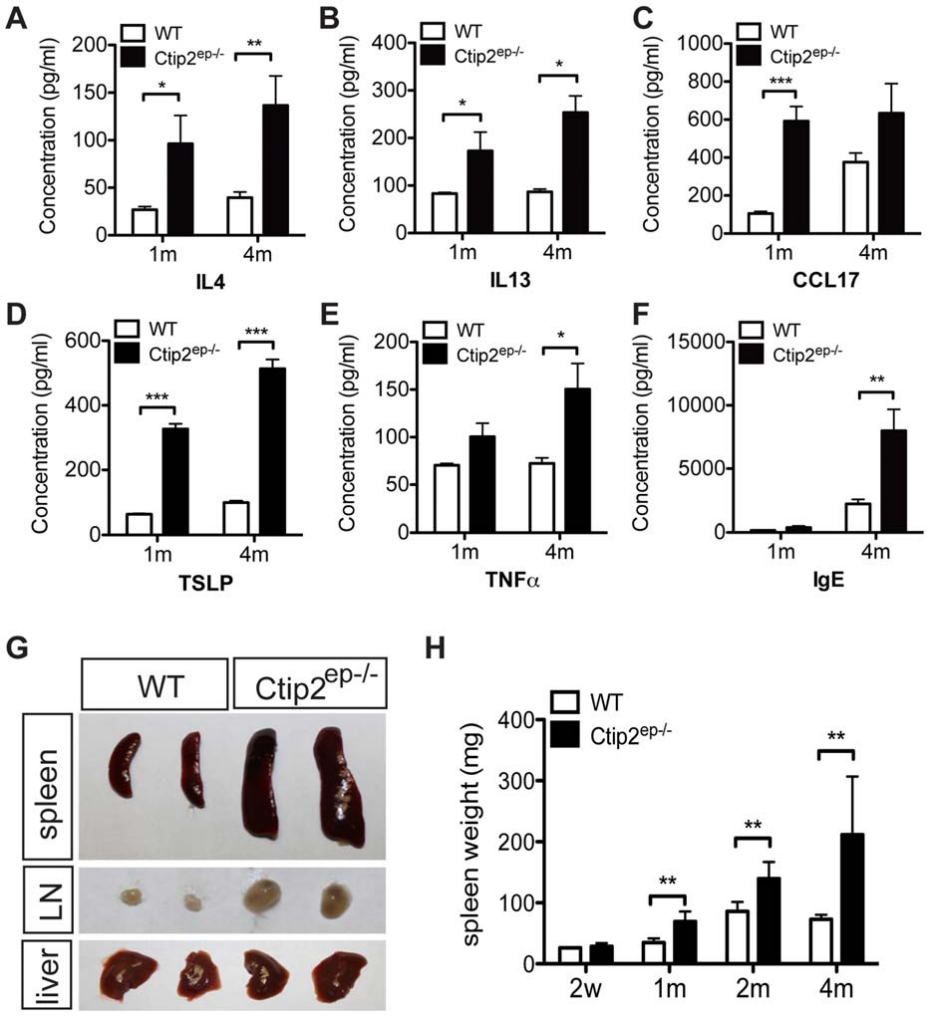
Systemic immunological abnormalities in Ctip2^ep−/−^ adult mice. Serum concentration of (**A**) IL4, (**B**) IL13, (**C**) CCL17, (**D**) TSLP, (**E**) TNFα and (**F**) IgE was measured by ELISA. (**G**) Gross morphological comparison of spleen, lymph node (LN) and liver in 4-months-old WT and Ctip2^ep−/−^ mice. (**H**) Spleen weight of WT and Ctip2^ep−/−^ mice at different months of age is indicated. Statistical analyses were performed by student's unpaired t-test using GraphPad Prism software; * P<0.05, ** P<0.01, *** P<0.005.

We hypothesized that chronic skin inflammation developed in Ctip2^ep−/−^ mice may trigger systemic immune responses in the mutant skin. To test that we determined blood plasma concentrations of IgE as well as various Th2-type cytokines/chemokines (IL-4, IL-13, CCL17 and TSLP) and Th1-type cytokine TNFα from 1-month and 4-months-old mice by ELISA (Figure 5A–F). Plasma level for all of the tested Th2 cytokines, were observed to be higher in both 1-month and 4-month old mutant mice (Figure 5A–E), including IL4 and the related IL-13 (Figure 5A, B), CCL17 (Figure 5C) and TSLP (Figure 5D). In contrast, plasma level of Th1 cytokine TNFα was higher only at 4-month but not in 1-month old mutant mice (Figure 5E). While circulating IgE levels were elevated in both wild-type and mutant mice at four months of age, IgE levels in mutant mice were 2.5 times greater than those in wildtype mice (Fig. 5F).

Ctip2^ep−/−^ mice exhibited inguinal lymphadenopathy and smaller convoluted thymus at four months of age (Figure 5G, data not shown). The mutant mice displayed progressive splenomegaly starting from one month of age, with a 2-fold increase in spleen weight at the age of four months (Figure 5G–H). Histological studies of the spleens revealed an increased cell infiltration in the 4-month-old mutant spleen in comparison with wildtype (Figure S6 A, D, G, J). 4 month-old Ctip2^ep−/−^ mice exhibited increased eosinophil infiltration in the spleen and lymph node compared with wildtype (Figure S6, B, E, H, K). Very few mast cells were observed in both wildtype and mutant mice spleens and the number of mast cells was larger in the lymph node, indistinguishable between 4 month-old wildtype and mutant mice (Figure S6, C, F, I, L). These results clearly indicate that Ctip2^ep−/−^ mice display a systemic immune response that may be primarily driven by TSLP expression from keratinocytes.

## Discussion

Epidermal keratinocytes have long been suspected to play a crucial role in initiating and directing the immune response in inflammatory skin diseases, such as AD. In the present report we have shown that skin of adult Ctip2^ep−/−^ mice, with an epidermal-specific deletion of the gene encoding Ctip2, display histological and cellular features that are typical of human AD. First, Ctip2^ep−/−^ mice develop skin lesions, alopecia and pruritus, which are major clinical features in patients afflicted with AD [4], [33], [34]. Second, Ctip2^ep−/−^ mice exhibit epidermal hyperplasia that is characterized by increased epidermal proliferation, similar to what has been observed in AD patients [2]. Third, the skin of Ctip2^ep−/−^ mice is characterized by a dramatic upregulation of Th2-type cytokines and chemokines, including TSLP, CCL17, IL13 and IL4, all of which were induced early in Ctip2^ep−/−^ epidermis, and are crucially involved in the initiation of pathogenesis in human AD [3], [7], [35]. Finally, Ctip2^ep−/−^ adult mice exhibit Th2-like systemic immune syndrome that is similar to that found in most AD patients [36]. Based on the above phenotypic spectrum of Ctip2^ep−/−^ adult mice, it appears that this line may serve as a useful model for the study of human AD.

During development, selective ablation of Ctip2 in the epidermis reduced epidermal proliferation and impaired terminal differentiation of keratinocytes [25]. Isolated Ctip2-null keratinocytes also exhibit severe proliferation and differentiation defects in culture [26]. To our surprise, in the postnatal stages, a hyperplastic epidermis and a significantly higher percentage of Ki67-positive cells were observed in the Ctip2^ep−/−^ skin as early as 1 week after birth (Figure 1). Elevated proliferation could be secondary to impaired epidermal barrier functions caused by Ctip2 ablation. Previous studies have established that hyperproliferation and acanthosis (thickened epidermis) are compensatory responses and secondary to impaired epidermal barrier [37]–[40]. It is also possible that impaired skin barrier in Ctip2^ep−/−^ mice skin could be partially due to deficiency in tight junctions. That possibility was ruled out since immuno-histochemical staining of dorsal skin exhibited no significant difference in tight junction protein expression including beta-catenin, E-cadherin, claudin-1 and claudin-4 between wildtype and mutant mice (Figure S4B–E).

A number of candidate genes have been identified in association with AD, however, many of these linkages have been called into question due to insufficiently powered studies and/or heterogeneity of the disease [33], [34]. In independent studies, several genes such as Filaggrin (FLG), trans-acting T-cell-specific transcription factor (GATA3) and IL4 has been reported to be involved in AD pathogenesis [41]. FLG deficiency alone decreases stratum corneum hydration and leads to increased TEWL, and has been correlated with AD in the highest number of studies [41], [42]. It is worth mentioning that skin barrier deficiency has also been reported in AD patients without FLG mutation(s) or loss of expression [43] suggesting existence of different subtypes of AD in humans. We have shown previously that Ctip2 deficiency is associated with impaired terminal differentiation and decreased FLG expression during development [25]. Indeed, we have observed a ∼50% reduction of FLG expression in the epidermis of Ctip2^ep−/−^ skin compared to that of wildtype littermates around 1 week of age (Figure S1F). However, no significant difference in level of FLG expression was observed between the wildtype and mutant epidermis at later timpoints from 2 weeks to 4 months of age, possibly due to a compensatory upregulation of other related factors such as Ctip1 (Figure 1E). The lack of loss-of-FLG expression observed in Ctip2^ep−/−^ mice at later stages indicates possible involvement of other factors to modulate barrier integrity.

It appears that during adulthood, the requirement of Ctip2 for epidermal proliferation and differentiation is compensated by upregulation of a Ctip2-independent growth pathway. The increase of epidermal proliferation may also be secondary to the inflammatory phenotype together with defects in barrier function. The role of Th2 cytokines in AD skin lesion is to promote the recruitment, proliferation and development of Th2-type immune cells to lesional site, whereas their direct roles in keratinocyte proliferation still remain largely unknown. Activation of TSLP signaling in dendritic cells and T cells is known to activate multiple growth pathways, including STATs, Src kinases, PI3K and ERK1/2 [44]–[48]. It is possible that inflammatory cytokines, such as TSLP may contribute to the hyperproliferative response of Ctip2^ep−/−^ adult epidermis.

TSLP, a keratinocyte-derived triad of cytokines, has been studied intensively recently in the pathogenesis of allergic inflammation and subsequent development of AD and other immune disorder [31], [49], [50]. It is recognized as the master switch for AD and other atopic diseases, such as asthma, as it plays a critical role to induce the Th2-type allergic inflammation in AD pathogenesis [31]. Keratinocyte-derived TSLP participates in the inflammatory cascade by activation of other immune cells, such as dendritic cells and mast cells, and subsequent stimulation of release of Th2-related cytokines/chemokines from these immune cells, eventually initiate inflammatory a Th2 lymphocyte response [9], [49], [51]. In the present study, we have observed that TSLP was the earliest known cytokine induced in Ctip2^ep−/−^ mice and this induction was maintained in the mutants at all postnatal stages of development. The induction of TSLP was mainly restricted to the epidermis, which correlates well with epithelial cells of the keratinocyte lineage as major producers of TSLP in AD patients (see Figures 4F and S4A) [49]. Hence, induction of TSLP in Ctip2 mutant keratinocytes could be a trigger factor that initiates and directs the Th2-type inflammatory cascade observed in Ctip2^ep−/−^ skin.

Transgenic mice over-expressing TSLP in keratinocytes develop AD-like symptoms, indicating that TSLP expression is sufficient to initiate AD-like inflammatory responses [50]. Therefore, it remains critical to address upstream triggers of TSLP expression and how TSLP is regulated before and during AD pathogenesis. Putative NF-κB response element has been detected in the mouse and human TSLP gene promoter and NF-κB regulated mouse and human TSLP gene expression in presence of inflammatory cytokines *in vitro* [13], [14], [52]–[54]. Putative AP-1 transcription factor binding motif was also detected on the promoter region of human TSLP gene [13], [14], [52]–[54]. Similarly, nuclear receptor (NR) RXR directly repressed TSLP transcription through the binding of RXR-VDR and RXR-RARγ heterodimer containing co-repressor complexes to the mouse TSLP promoter [13], [14], [52]–[54]. Previous studies have also identified presence of putative VDREs (DR3) and RAREs (DR2 and DR1) upstream of the human TSLP promoter [14]. Since we have observed Ctip2 recruitment around that region on the mTSLP promoter, it is possible that NRs and Ctip2 could be a part of the same transcriptional complex to regulate TSLP gene expression in mouse keratinocytes. However, additional recruitment of Ctip2 along with NRs on the further distal region of mouse TSLP gene promoter cannot be ruled out. Here, we have identified a novel transcriptional regulatory mechanism(s) of negative regulation of TSLP expression by Ctip2 in mouse epidermis. Ctip2 appears to interact directly with and repress expression of the TSLP promoter.

We have demonstrated a predominant upregulation of Th2 cytokines in the Ctip2^ep−/−^ skin at all timepoints, but a late surge of Th1 cytokines, including IFNγ and TNFα, was also observed in the mutant skin at 4 months (Figure S3N-O). Indeed, it has been demonstrated that Th2- and Th1 type cytokines both contribute to different stages of the development of skin lesions in AD patients [29]. Th2-cell subsets are specifically activated during the initiation phase, followed by the Th1-cell subset to maintain the persistence of the chronic inflammatory response. Our work confirms that Th2- and Th1-type immune responses are not mutually exclusive, especially at the chronic phage of the inflammatory disease. The interaction between different Th-cell subsets at the chronic stage may be a fruitful area for future investigation of AD pathogenesis.

Systemic immune response related to elevated expression of IL-4, IL13, CCL17 and IgE, observed in Ctip2^ep−/−^ mice is evidently Th2 cell-dependent and humoral in nature. IL-4, for example, induces differentiation of CD4^+^ T-cells into Th2 cells, and also inhibits production of Th1 cells [55]. Th2 cells secrete IL-13, which together with IL-4, initiates a B lymphocyte-directed, humoral response [55]. IL-4 also upregulates MHC class II expression and induces B-cell class switching to IgE [56]. Furthermore, TSLP stimulation of naïve CD4^+^ T-cells induces a specialized Th2 polarization, which results in production of downstream Th2-associated pro-inflammatory cytokines, such as IL-13, which carry out humoral immune processes [30]. Simultaneously, CCL17 is known to attract Th2 cells, thereby favoring humoral responses [57]. In summary, inflammation due to selective Ctip2 ablation in mice epidermis is not restricted to the site of deletion; rather Ctip2^ep−/−^ mice show a chronic secondary Th2-dependent humoral systemic inflammatory response that could possibly lead to inflammation of airway and pulmonary epithelium and asthma.

Our group has previously shown that Ctip2 expression was increased in samples from AD and ACD patients, and here we also show an increase of Ctip2 expression in the epidermis of another known mouse AD model (RXRα^ep−/−^; Figure S7A–B) [24]. Based on our study here, we hypothesize that induction of Ctip2 during AD progression may have a protective role, which is to suppress the inflammatory response by inhibiting TSLP expression. In contrast, the elevated proliferation in the Ctip2^ep−/−^ mice epidermis is likely due to compensatory responses and secondary to impaired epidermal barrier functions. Additional studies to determine TSLP level in AD and ACD patients with elevated Ctip2 expression will be useful to validate our hypothesis.

In summary, our study reveals a novel role(s) of keratinocytic Ctip2 in epidermal barrier maintenance and homeostasis, and inflammation in adult mice. Ctip2 clearly modulates AD-like responses within the skin, as well as systemic inflammatory responses. TSLP was strongly induced in Ctip2^ep−/−^ epidermis and likely causes increased dendritic cells infiltration into lymph nodes, in which these cells interact with CD4^+^ T cells to produce Th-2 inflammatory cytokines. Subsequently, other immune cells such as macrophages, leukocytes, mast cells and eosinophils could be attracted and recruited to the site of skin inflammation, which become disseminated, leading to elevated circulating levels of inflammatory mediators. This study identifies a new mediator, Ctip2, which may be implicated in the etiology of AD. However, several issues need to be resolved in the future. For example, it is presently unclear if a defective skin barrier precedes inflammation and enhanced cellular proliferation in the early stages of AD development. Second, the contributory role of TSLP in AD and the mechanistic basis of regulation of TSLP gene expression in mouse and human keratinocytes have not been fully elucidated. Finally, the role of Ctip1 in AD development in presence or absence of Ctip2 could shed further lights in AD pathogenesis and identify new avenues for therapeutic intervention. Our study highlights a central role of epidermal keratinocytes in initiating and shaping the immune response during the pathogenesis of AD-like skin diseases in mice.

## Materials and Methods

### Mice

The function of Ctip2 in adult mice skin homeostasis was characterized using Ctip2^ep−/−^ mice that selectively lacked Ctip2 in epidermal keratinocytes after Cre-recombinase mediated deletion of Ctip2 gene in keratinocytes using K14-Cre deleter mice [25], [27], [28]. Ctip2 floxed mice (Ctip2 ^L2/L2^, wildtype) were used as controls [25], [28]. 8 to 10 mice from multiple litters were used in each group at each time point. Mice were maintained in a temperature/humidity-controlled facility with a 12-hour light/dark cycle. Animal protocol was approved by Oregon State University Institutional Animal Care and Use Committee (IACUC), under permit number 4300.

### Transepidermal water loss (TEWL) measurement

Transepidermal water loss was measured using the calibrated Tewameter TM300 with Multi Probe Adapter (CK electronic GmbH, Köln, Germany) in accordance with manufacture operating instructions. Data was expressed in g/m^2^ h, and represents the mean ± S.E.M. for 8 independent animals (10 measurements each animal) of each genotype. Statistical analysis was performed using Graphpad Prism software with student's unpaired t-test.

### Histological analysis

Skin biopsies were fixed in 4% paraformaldehyde overnight and embedded in paraffin blocks. 5 mm paraffin sections were sectioned using a Leica RM2255 microtome (Bannockburn, IL). Hematoxylin and eosin staining was performed according to general protocols [58].

Combined eosinophil/mast cell stain (C.E.M) was performed using a commercial kit according to the manufacturer's protocol (American MasterTech, Lodi, CA). Briefly, slides were deparaffinized with xylene and hydrated through alcohols. After washed in running tap water, slides were placed in astra blue staining solution for 30 minutes and rinsed in tap water. Slides were put in vital new red staining solution for another 30 minutes and rinsed with tap water. After that, slides were placed in modified Mayer's hematoxylin for 15–30 seconds and rinsed again with tap water for 2 minutes. Finally, dehydration of slides was performed with 3 changes of absolute alcohol and xylene before mounting. Mast cells showed bright blue color while eosinophils were red. Nuclei were stained blue.

Toluidine blue staining was performed as described [13]. Toluidine blue working solution was prepared by mixing 5 ml 1% toluidine blue EtOH solution (Sigma-Aldrich, St. Louis, MO) with 45 ml 1% sodium chloride (pH 2.3). Slides were deparaffinized and hydrated in distilled water. Sections were stained with toluidine blue working solution (pH 2.0–2.5) for 2–3 minutes before washed in 3 changes of distilled water. Slides were dehydrated through alcohols and xylenes and mounted with resinous mounting medium.

### Immunohistochemistry

IHC staining of paraffin and frozen sections was described previously [58], [59]. In brief, paraffin sections were deparaffinized and antigen retrieval was performed with pH 6.0 citrate buffer and 95–100°C for 20 minutes and cooled down. For frozen sections, slides were fixed with cold acetone for 20 minutes and allowed for air dry. Slides were washed with 0.1% PBST and blocked with 10% normal goat serum (Vector Laboratories, Builingame, CA) for 30 minutes before incubated with primary antibodies overnight at 4°C. Fluorescently labeled secondary antibodies were applied to slides for an hour at room temperature. Nuclei were visualized with DAPI. After the final washes, slides were dehydrated and mounted. Images were captured at ×20 magnification using a Carl Zeiss Axio Imager Z1 fluorescent microscope and AxioCam camera. Data were analyzed and quantified using Adobe Photoshop and ImageJ software. Multiple IHC fields were randomly chosen and 10–15 such fields per slide were counted independently in a double-blinded manner. All experiments in each category were repeated in triplicates.

The concentrations of antibodies used were as follows: Ki67 (Abcam, Cambridge, MA, 1∶500), K14 (Abcam, 1∶1000), K10 (Abcam, 1∶1000), Filaggrin (Abcam, 1∶1000), Loricrin (Abcam, 1∶500), F4/80 (Biolegend, San Diego, CA, 1∶250), CD11b (Abcam, 1∶100), CD45 (Abcam, 1∶500), Ly6g (Abcam, 1∶250), CD11c (BD Pharmingen, San Jose, CA, 1∶100), RXRα (Santa Cruz Biotechnology, Santa Cruz, CA, 1∶1000), Notch1 (Cell Signaling technology, Danvers, MA, 1∶500), Ctip2 (Abcam, 1∶300), Claudin4 (Abcam, 1∶50), Claudin1 (Abcam, 1∶100), E-cadherin (Cell Signaling, Danvers, MA, 1∶200), b-Catenin (Cell Signaling, 1∶50), TSLP (Invitrogen, 1∶100), Cy3 goat anti rabbit (Jackson ImmunoResearch Laboratories Inc., West Grove, PA, 1∶500), Cy3 goat anti rat (Jackson ImmunoResearch, 1∶500), Cy2 goat anti rat (Jackson ImmunoResearch, 1∶500), Cy3 goat anti mouse (Jackson ImmunoResearch, 1∶1000).

### RT-qPCR

RNA extraction and cDNA preparation were performed as described [60]. Real-time PCR was performed on an ABI 7500 Real-Time PCR system using SYBR Green methodology using specific sets of primers as indicated in Table S1 [60], [61]. Relative gene expression analysis of the RT-qPCR data was performed using HPRT as an internal control. All assays were performed in triplicates.

### ELISA

ELISA kits for detecting mouse IL-4, IL-13, TNFα and TSLP were obtained from eBioscience, Inc. (San Diego, CA, USA). Mouse IgE ELISA kit was procured from Bethyl Laboratories, Inc. (Montgomery, TX, USA), while CCL17 immunoassay kit was purchased from R&D Systems, Inc. (Minneapolis, MN, USA).

1-month and 4-month-old mice (6 wildtypes and 6 Ctip2^ep−/−^ from each age group) were selected for studying secondary systemic response to Ctip2^ep−/−^ genotype. Blood plasma was isolated from the mice following the method of Argmann and Auwerx [62]. ELISA was performed for estimation of the levels of IL-4, IL-13, IgE, CCL17, TNFα and TSLP using kits following the respective manufacturer's instructions. All samples were assayed in triplicates.

### Chromatin immunoprecipitation (ChIP)

ChIP assay was performed according to Hyter *et al.*, 2010 with minor modifications [63]. Briefly, mouse epidermal keratinocytes from 1-day postnatal wildtype mice skin were isolated and fixed in 1% formaldehyde. Cell lysate from 3×10^6^ keratinocytes (each ChIP) were obtained after incubation with hypotonic buffer (10 mM Tris/HCl, pH 7.5, 10 mM NaCl, 3 mM MgCl_2_ and 0.5% NP40). Nuclei were suspended in ChIP sonication buffer (50 mM Hepes, pH 7.9, 150 mM NaCl, 1 mM EDTA, 1% Triton X-100, 0.1% SDS, 0.1% sodium deoxycholate) with 0.2% SDS. Nuclei were sonicated and immunoprecipitated with 2 µg anti-Ctip2 antibody (Abcam, Cambridge, MA) overnight at 4°C. Rat IgG was used as a control. 25 ml pre-equilibrated protein G beads (Invitrogen, Carlsbad, CA) were added to each ChIP and incubated for four hours at 4°C. After washing twice with ChIP sonication buffer, buffer A (50 mM Hepes, 500 mM NaCl, 1 mM EDTA, 1% Triton X-100, 0.1% SDS and 0.1% sodium deoxycholate), buffer B (20 mM Tris/HCl, pH 8.0, 1 mM EDTA, 250 mM LiCl, 0.5% NP40 and 0.5% sodium deoxycholate) and once with TE buffer, DNA was uncrosslinked at 65°C overnight and purified by QIAquick PCR Purification Kit (Qiagen, Germantown, MD). All the buffers used in the study were added with PMSF and protease inhibitors before use. DNA was amplified using ABI-7500 Real-time PCR machine with specific primers indicated in Table S1. Primers were designed from proximal (−200 bp) and distal (−1 kb) promoter regions of TSLP to study the possible interaction between Ctip2 and TSLP, and primers selected from 3′-UTR region was used as a control.

### Western blotting

Protein was extracted from mouse epidermis and western blotting was performed according to previous reports using specific antibodies against filaggrin (Abcam,1∶1000) and loricrin (Abcam, 1∶1000) [58]. Western blotting represents the results from three separate experiments performed in triplicate.

### Statistics

Statistical significance of differences between groups was analyzed using GraphPad Prism software using student's unpaired *t*-test. All statistical analyses were performed in a double-blinded manner.

## Supporting Information

Figure S1 **Characterization of epidermal proliferation and differentiation in Ctip2^ep−/−^ mice.** (**A**) Measurement of epidermal thickness of WT and Ctip2^ep−/−^ mice skin. (**B**) Measurement of trans-epidermal water loss (TEWL) from dorsal skin of wildtype, lesional Ctip2^ep−/−^ mice and non-lesional Ctip2^ep−/−^ mice at 4 month. (**C**) Epidermal percent Ki67 positive cells in dorsal skin sections of WT and mutant mice. Statistical analyses were performed by student's unpaired t-test using GraphPad Prism software; * P<0.05, ** P<0.005, *** P<0.0001. Immunohistochemical staining of dorsal skin biopsies from WT and Ctip2^ep−/−^ mice was performed with antibodies directed against (**D**) K14 and (**E**) K10 (all in red). All sections were counterstained with DAPI (blue). Scale bar (in D and E): 100 µm. Epidermis (E) and dermis (D) are indicated. (**F**) Quantitative RT-PCR (RT-qPCR) analyses of filaggrin in the dorsal skin of 1-week and 2-week- old wild type (WT) and Ctip2^ep−/−^ mice using specific primers as indicated in Table S1. ** P<0.005. All values represent relative transcript level after normalization with HPRT transcripts. (**G**) Immunoblotting analysis of filaggrin (FLG) and loricrin (LOR) in the skin of 2-week and 4-month-old wild type (WT) and Ctip2^ep−/−^ mice. β-actin is used as an internal control. Statistical analyses were performed by student's unpaired t-test using GraphPad Prism software; ** P<0.01, *** P<0.001.
(TIF)

Figure S2 **Characterization of inflammatory cell infiltrates in dorsal skin of WT and Ctip2^ep−/−^ adult mice.** (**A**) Toluidine blue stained dorsal paraffin skin sections of WT and Ctip2^ep−/−^ mice. Mast cells stain intensive blue color. Scale bar: 100 µm. (**B**) Immunohistochemical staining of dorsal skin biopsies with antibody against CD45 (green). Scale bar: 100 µm. (**C**) Percent CD45 positive cells in the dermis of WT and mutant skin. Statistical analyses were performed by student's unpaired t-test using GraphPad Prism software; * P<0.05. Scale bar: 100 µm. (**D**) Immunohistochemical staining of CD11c (red) in dorsal skin of WT and Ctip2^ep−/−^ mice. Scale bar: 50 µm. All sections (in B & D) were counterstained with DAPI (blue).
(TIF)

Figure S3 **Relative expression levels of cytokines and chemokines in WT and Ctip2^ep−/−^ skin.** The expression level of (**A**) IL10, (**B**) IL1α, (**C**) IL2, (**D**) IL12a, (**E**) IL12b, (**F**) IL17a, (**G**) IL18, (**H**) IL23, (**I**) CCL20, (**J**) CCL22, (**K**) CXCL10, (**L**) CCL3, (**M**) CXCL2, (**N**) IFNγ and (**O**) TNFα was studied with RT-qPCR in 1-month and 4-month-old wildtype and Ctip2^ep−/−^ dorsal skin. Values represent relative transcript level after normalization with HPRT transcripts. Statistical analyses were performed by student's unpaired t-test using GraphPad Prism software; * P<0.05, ** P<0.01, *** P<0.001.
(TIF)

Figure S4 **Characterization of TSLP and tight junction proteins in dorsal skin of 1-month and 4-month-old WT and in Ctip2^ep−/−^ adult mice.** Immunohistochemical staining of dorsal skin biopsies from WT and Ctip2^ep−/−^ mice were performed with specific antibodies against (**A**) TSLP; (**B**) β-catenin, (**C**) E-cadherin; (**D**) Claudin-1 and (**E**) Claudin-4 (all in red). All sections were counterstained with DAPI (blue). Scale bar: 100 µm.
(TIF)

Figure S5 **Relative expression levels of RXRα and genes involved in Notch signaling pathway.** The expression level of (**A**) Notch1, (**B**) Notch2, (**C**) Rbp-j and (**D**) RXRα was studied with RT-qPCR in 1-month and 4-month-old wildtype and Ctip2^ep−/−^ dorsal skin. Values represent relative transcript level after normalization with GAPDH transcripts. Statistical analyses were performed by student's unpaired t-test using GraphPad Prism software. (E) Immunohistochemical staining of dorsal skin biopsies with antibody against Notch1 (red) and (F) RXRα (red). Scale bar: 100 µm. All sections (in E & F) were counterstained with DAPI (blue).
(TIF)

Figure S6 **Immunological abnormalities of spleen and lymph node in Ctip2^ep−/−^ adult mice.** (**A, D**) Hemotoxylin & Eosin stained 5 µm thick paraffin sections from spleen of WT and Ctip2^ep−/−^ mice at 4 m. (**B, C, E, F**) C.E.M staining for eosinophils (pink) and mast cells (blue) in 4 month-old mice spleen sections. (**G, J**) Hemotoxylin & Eosin stained 5 µm thick paraffin sections of WT and Ctip2^ep−/−^ mice lymph node at 4 m. (**H, I, K, L**) C.E.M staining for eosinophils (pink) and mast cells (blue) in 4 month-old mice lymph node. Black arrows point to eosinophils. Scale bar: 50 µm.
(TIF)

Figure S7 **Expression of Ctip2 in RXRα^ep−/−^ mouse model.** (**A**) Immunohistochemical staining of dorsal skin biopsies with antibody against Ctip2 (red). Sections were counterstained with DAPI (blue). NE, normal epidermis; HPE, hyperfroliferative epidermis. Scale bar: 100 µm. (**B**) Bar graph indicates the percentage of Ctip2 positive cells in the dorsal skin of normal epidermis (NE) from the wildtype (WT) and the hyperproliferative epidermis (HPE) form RXRα**^ep−/−^** mice. Statistical analyses were performed by student's unpaired t-test using GraphPad Prism software; ** P<0.01. All experiments were performed in triplicates.
(TIF)

Table S1 List of primers used for RT-qPCR.
(DOCX)
