# Supplementary figures and images for: Selective Ablation of Ctip2/Bcl11b in Epidermal Keratinocytes Triggers Atopic Dermatitis-Like Skin Inflammatory Responses in Adult Mice
Source: PLoS One. 2012 Dec 20;7(12):e51262. doi: 10.1371/journal.pone.0051262 (PMC3527437; doi:10.1371/journal.pone.0051262)

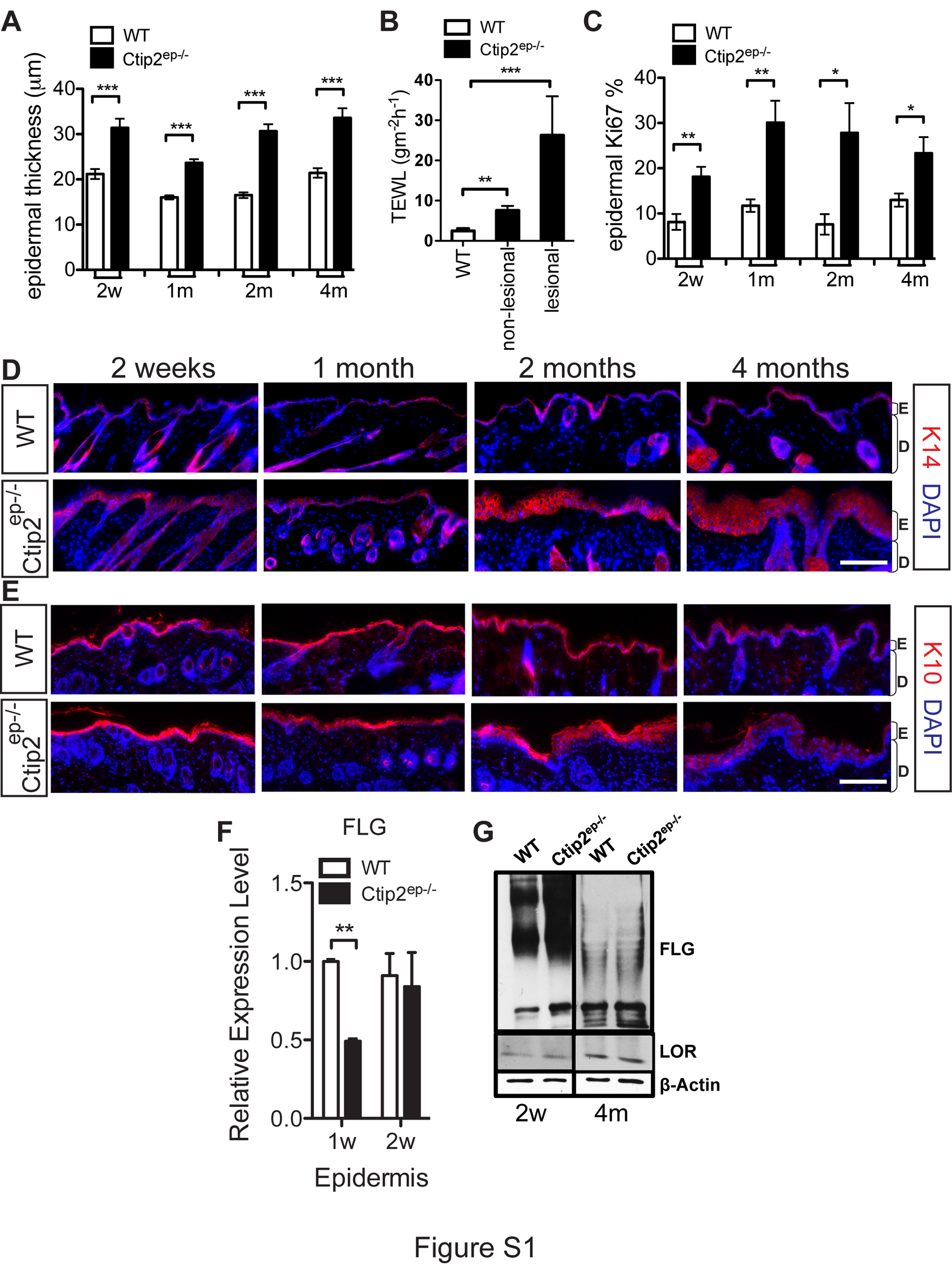

Supplement: Figure S1 — Characterization of epidermal proliferation and differentiation in Ctip2ep−/− mice. (A) Measurement of epidermal thickness of WT and Ctip2ep−/− mice skin. (B) Measurement of trans-epidermal water loss (TEWL) from dorsal skin of wildtype, lesional Ctip2ep−/− mice and non-lesional Ctip2ep−/− mice at 4 month. (C) Epidermal percent Ki67 positive cells in dorsal skin sections of WT and mutant mice. Statistical analyses were performed by student's unpaired t-test using GraphPad Prism software; * P<0.05, ** P<0.005, *** P<0.0001. Immunohistochemical staining of dorsal skin biopsies from WT and Ctip2ep−/− mice was performed with antibodies directed against (D) K14 and (E) K10 (all in red). All sections were counterstained with DAPI (blue). Scale bar (in D and E): 100 µm. Epidermis (E) and dermis (D) are indicated. (F) Quantitative RT-PCR (RT-qPCR) analyses of filaggrin in the dorsal skin of 1-week and 2-week- old wild type (WT) and Ctip2ep−/− mice using specific primers as indicated in Table S1. ** P<0.005. All values represent relative transcript level after normalization with HPRT transcripts. (G) Immunoblotting analysis of filaggrin (FLG) and loricrin (LOR) in the skin of 2-week and 4-month-old wild type (WT) and Ctip2ep−/− mice. β-actin is used as an internal control. Statistical analyses were performed by student's unpaired t-test using GraphPad Prism software; ** P<0.01, *** P<0.001. (TIF) [file pone.0051262.s001.tif]

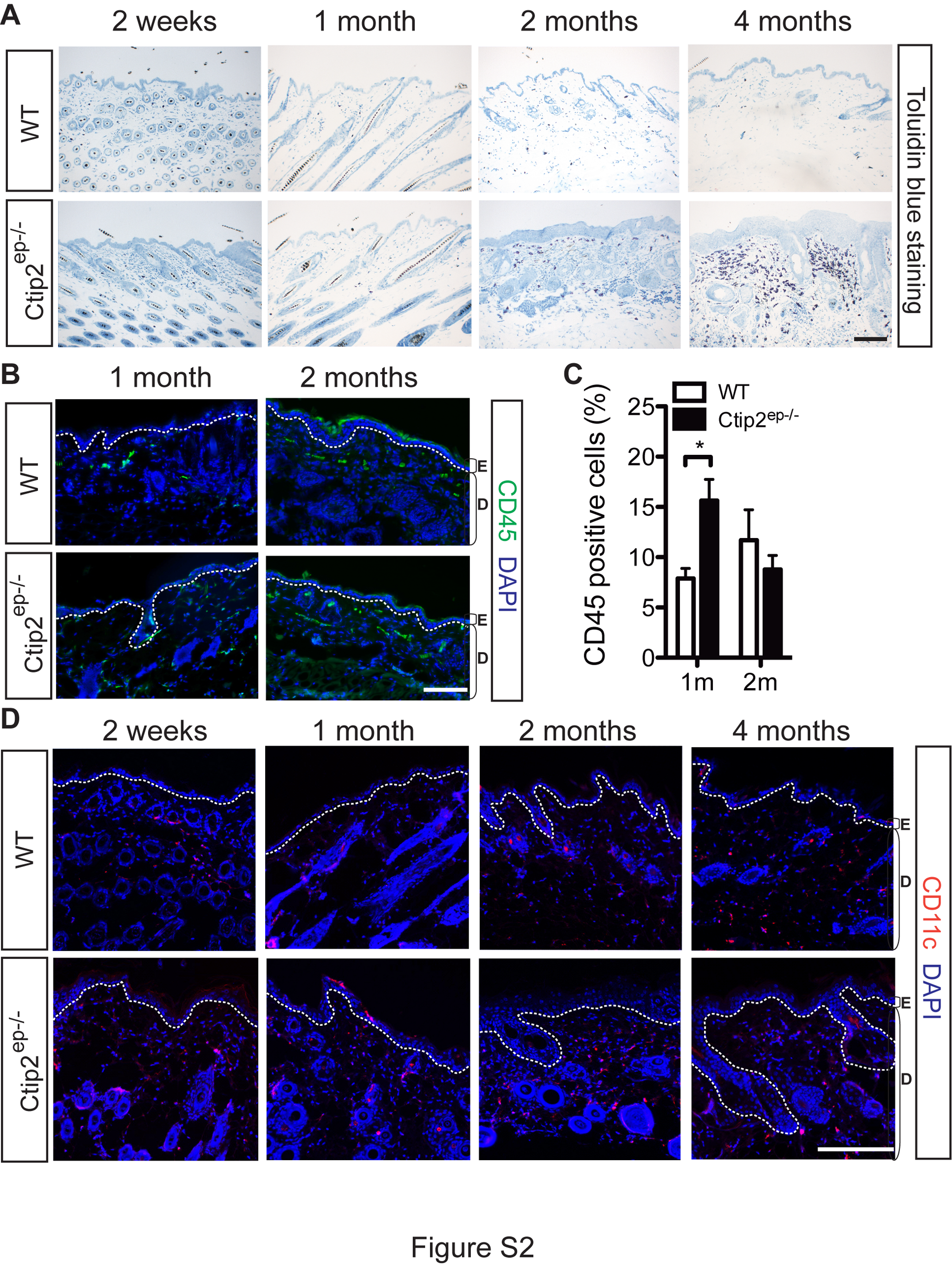

Supplement: Figure S2 — Characterization of inflammatory cell infiltrates in dorsal skin of WT and Ctip2ep−/− adult mice. (A) Toluidine blue stained dorsal paraffin skin sections of WT and Ctip2ep−/− mice. Mast cells stain intensive blue color. Scale bar: 100 µm. (B) Immunohistochemical staining of dorsal skin biopsies with antibody against CD45 (green). Scale bar: 100 µm. (C) Percent CD45 positive cells in the dermis of WT and mutant skin. Statistical analyses were performed by student's unpaired t-test using GraphPad Prism software; * P<0.05. Scale bar: 100 µm. (D) Immunohistochemical staining of CD11c (red) in dorsal skin of WT and Ctip2ep−/− mice. Scale bar: 50 µm. All sections (in B & D) were counterstained with DAPI (blue). (TIF) [file pone.0051262.s002.tif]

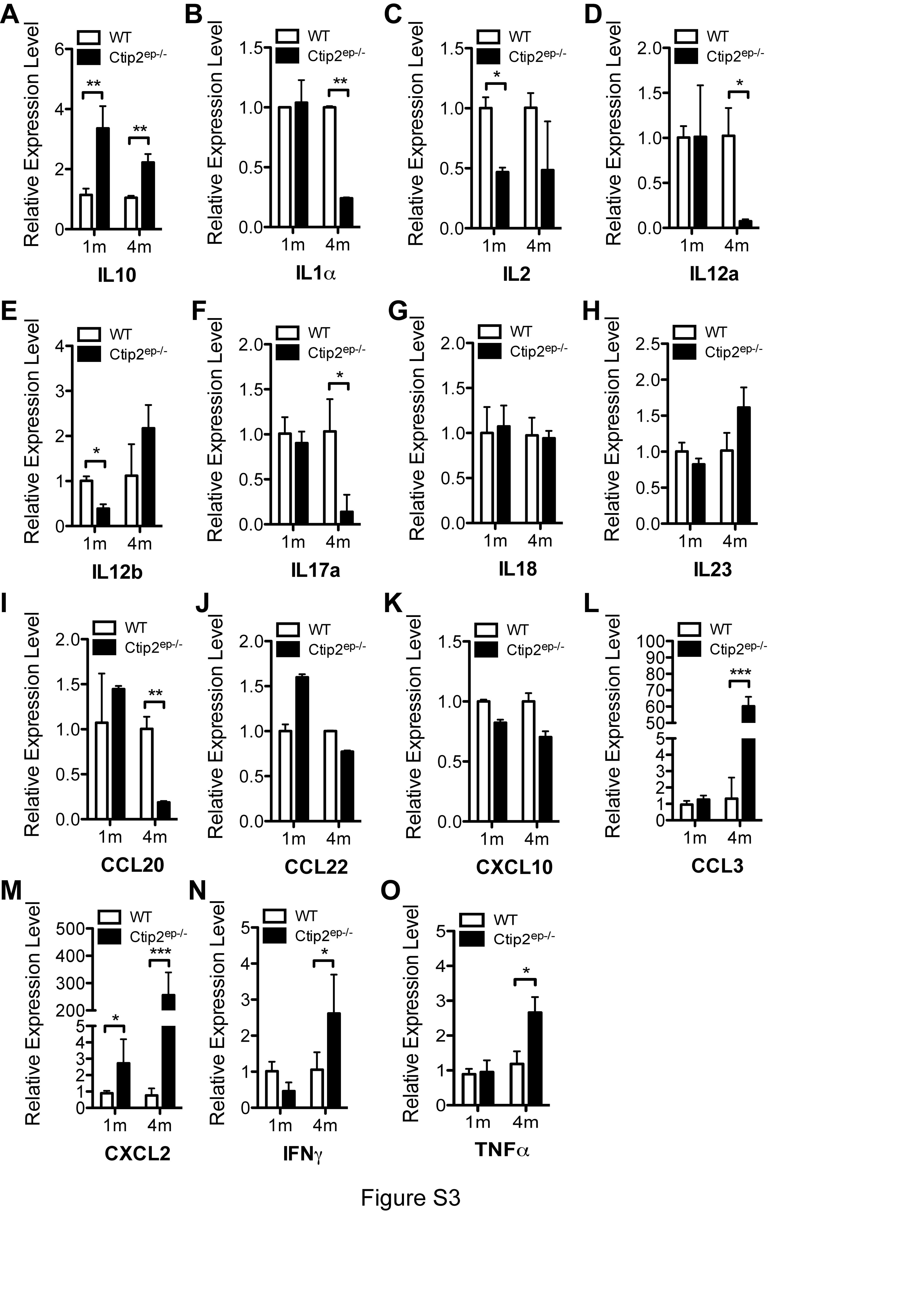

Supplement: Figure S3 — Relative expression levels of cytokines and chemokines in WT and Ctip2ep−/− skin. The expression level of (A) IL10, (B) IL1α, (C) IL2, (D) IL12a, (E) IL12b, (F) IL17a, (G) IL18, (H) IL23, (I) CCL20, (J) CCL22, (K) CXCL10, (L) CCL3, (M) CXCL2, (N) IFNγ and (O) TNFα was studied with RT-qPCR in 1-month and 4-month-old wildtype and Ctip2ep−/− dorsal skin. Values represent relative transcript level after normalization with HPRT transcripts. Statistical analyses were performed by student's unpaired t-test using GraphPad Prism software; * P<0.05, ** P<0.01, *** P<0.001. (TIF) [file pone.0051262.s003.tif]

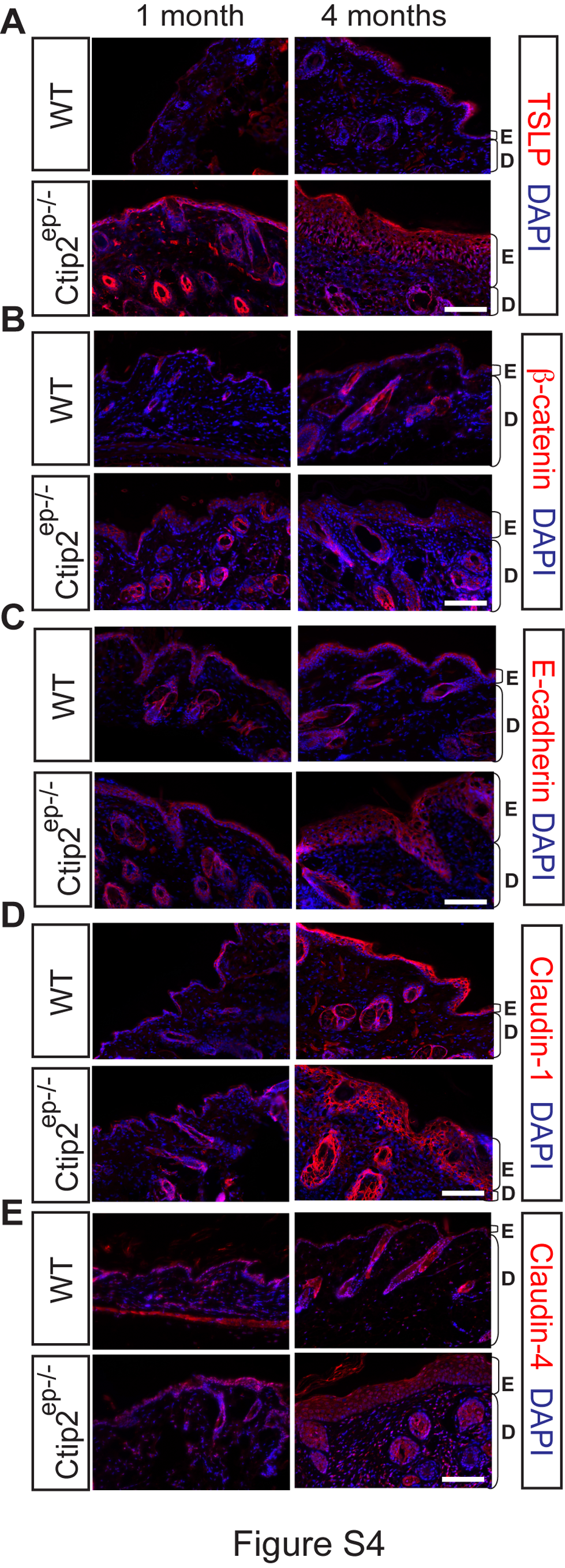

Supplement: Figure S4 — Characterization of TSLP and tight junction proteins in dorsal skin of 1-month and 4-month-old WT and in Ctip2ep−/− adult mice. Immunohistochemical staining of dorsal skin biopsies from WT and Ctip2ep−/− mice were performed with specific antibodies against (A) TSLP; (B) β-catenin, (C) E-cadherin; (D) Claudin-1 and (E) Claudin-4 (all in red). All sections were counterstained with DAPI (blue). Scale bar: 100 µm. (TIF) [file pone.0051262.s004.tif]

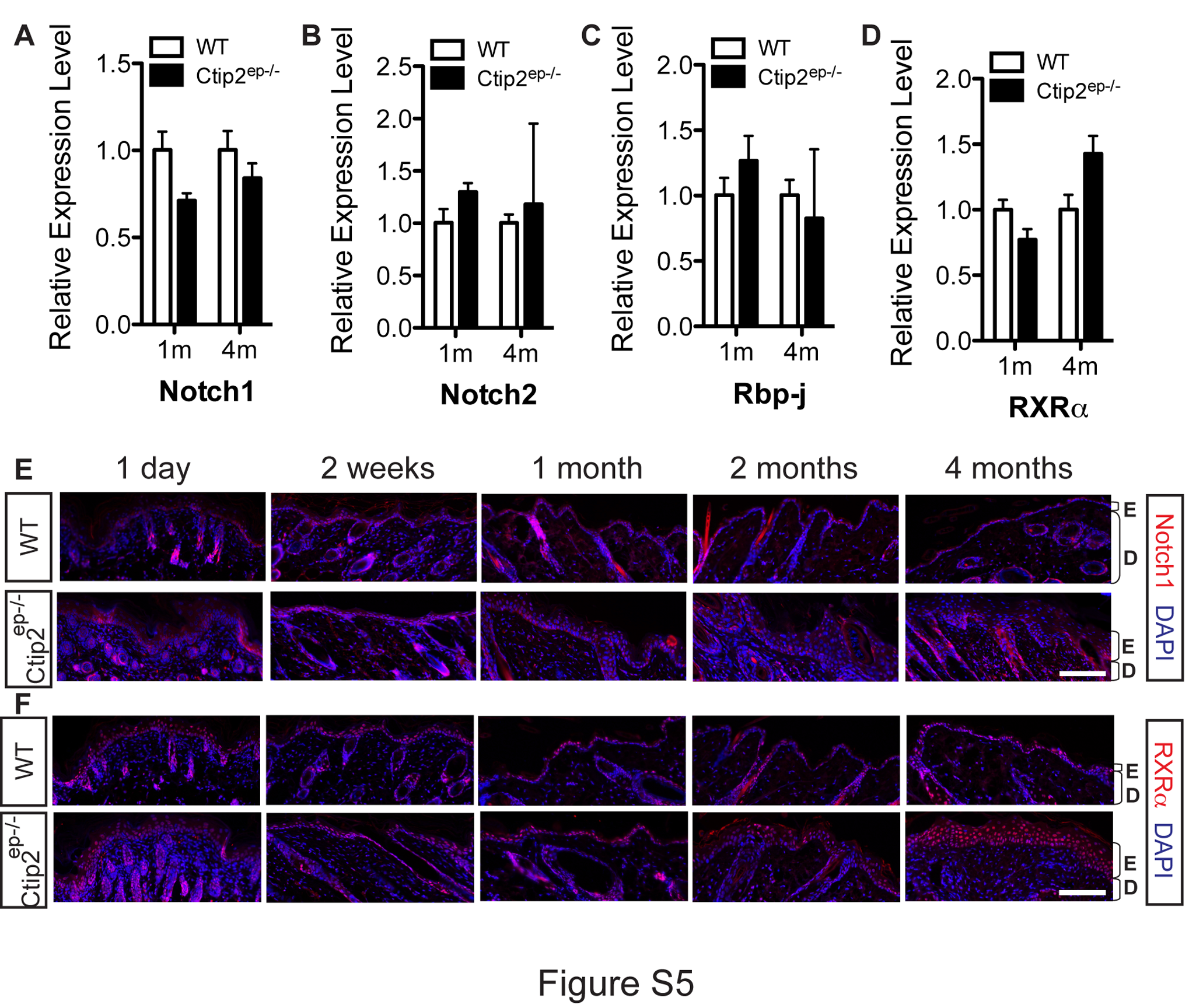

Supplement: Figure S5 — Relative expression levels of RXRα and genes involved in Notch signaling pathway. The expression level of (A) Notch1, (B) Notch2, (C) Rbp-j and (D) RXRα was studied with RT-qPCR in 1-month and 4-month-old wildtype and Ctip2ep−/− dorsal skin. Values represent relative transcript level after normalization with GAPDH transcripts. Statistical analyses were performed by student's unpaired t-test using GraphPad Prism software. (E) Immunohistochemical staining of dorsal skin biopsies with antibody against Notch1 (red) and (F) RXRα (red). Scale bar: 100 µm. All sections (in E & F) were counterstained with DAPI (blue). (TIF) [file pone.0051262.s005.tif]

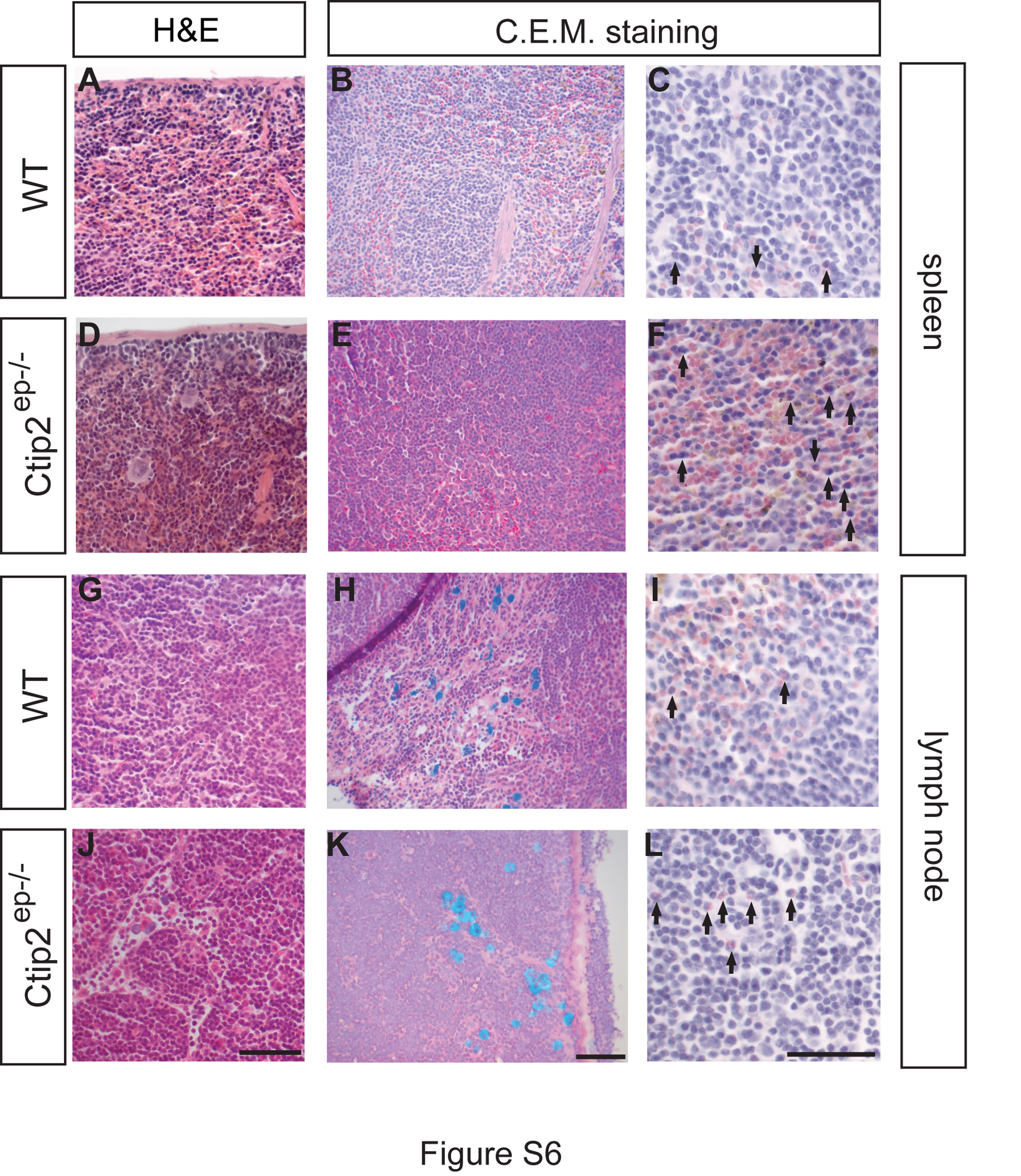

Supplement: Figure S6 — Immunological abnormalities of spleen and lymph node in Ctip2ep−/− adult mice. (A, D) Hemotoxylin & Eosin stained 5 µm thick paraffin sections from spleen of WT and Ctip2ep−/− mice at 4 m. (B, C, E, F) C.E.M staining for eosinophils (pink) and mast cells (blue) in 4 month-old mice spleen sections. (G, J) Hemotoxylin & Eosin stained 5 µm thick paraffin sections of WT and Ctip2ep−/− mice lymph node at 4 m. (H, I, K, L) C.E.M staining for eosinophils (pink) and mast cells (blue) in 4 month-old mice lymph node. Black arrows point to eosinophils. Scale bar: 50 µm. (TIF) [file pone.0051262.s006.tif]

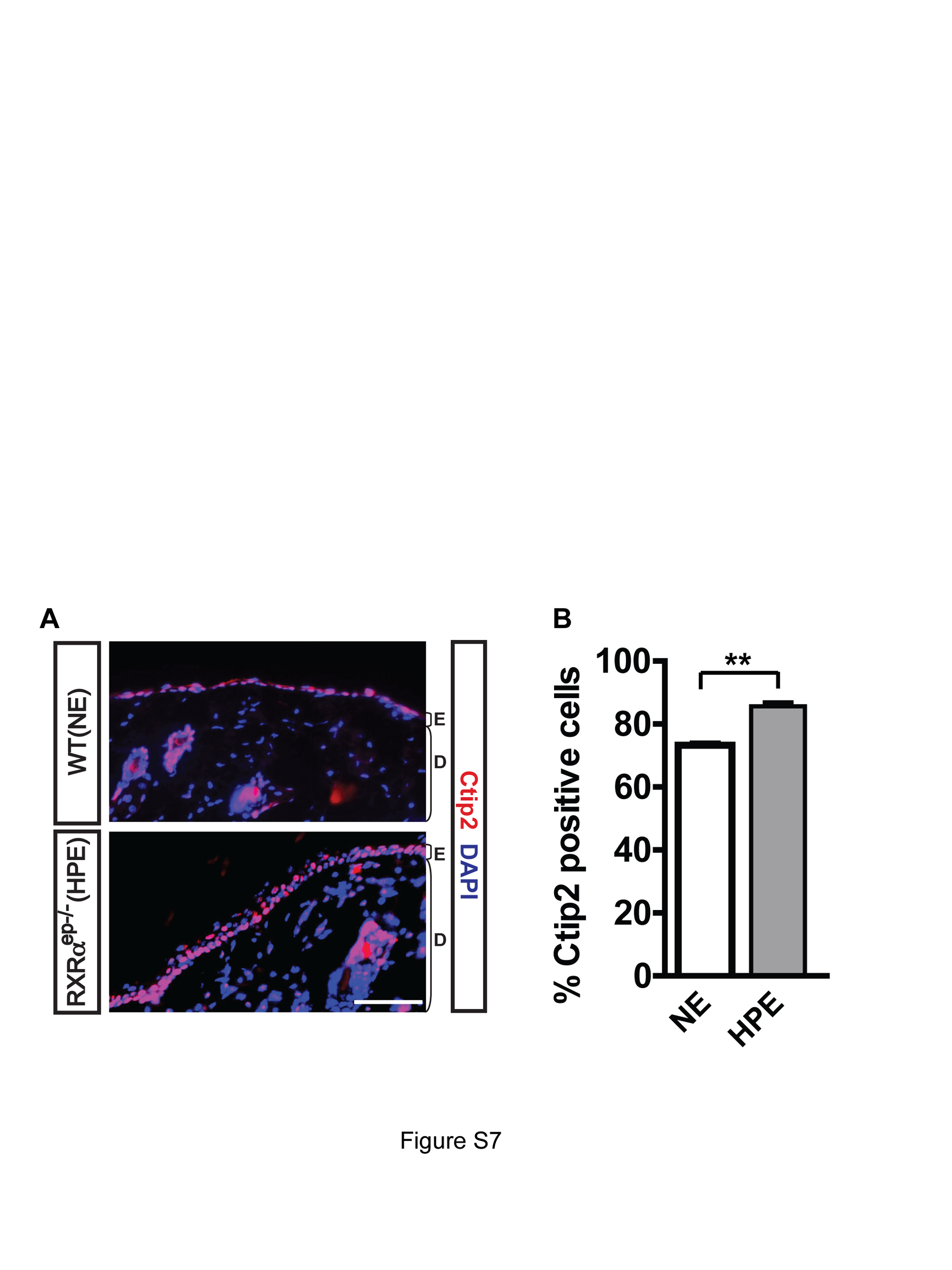

Supplement: Figure S7 — Expression of Ctip2 in RXRαep−/− mouse model. (A) Immunohistochemical staining of dorsal skin biopsies with antibody against Ctip2 (red). Sections were counterstained with DAPI (blue). NE, normal epidermis; HPE, hyperfroliferative epidermis. Scale bar: 100 µm. (B) Bar graph indicates the percentage of Ctip2 positive cells in the dorsal skin of normal epidermis (NE) from the wildtype (WT) and the hyperproliferative epidermis (HPE) form RXRαep−/− mice. Statistical analyses were performed by student's unpaired t-test using GraphPad Prism software; ** P<0.01. All experiments were performed in triplicates. (TIF) [file pone.0051262.s007.tif]
