# Supplementary material for: Selective Ablation of Ctip2/Bcl11b in Epidermal Keratinocytes Triggers Atopic Dermatitis-Like Skin Inflammatory Responses in Adult Mice
Source: PLoS One. 2012 Dec 20;7(12):e51262. doi: 10.1371/journal.pone.0051262 (PMC3527437; doi:10.1371/journal.pone.0051262)
Supplement: Table S1 — List of primers used for RT-qPCR. (DOCX) [file pone.0051262.s008.docx]

**Table S1 List of primers used for RT-qPCR**

| **Gene** | **Strand** | **Primer sequence** |
| --- | --- | --- |
| CCL3 | forward | CTCCCAGCCAGGTGTCATTTT |
|  | reverse | CTTGGACCCAGGTCTCTTTGG |
| CCL11 | forward | TCCACAGCGCTTCTATTCCT |
|  | reverse | CTATGGCTTTCAGGGTGCAT |
| CCL17 | forward | TACCATGAGGTCACTTCAGATGC |
|  | reverse | GCACTCTCGGCCTACATTGG |
| CCL20 | forward | TCACCTCTGCAGCCAGGCAGA |
|  | reverse | TCTTAGGCTGAGGAGGTTCACAGC |
| CCL22 | forward | CCGTCACCCTCTGCCATCACG |
|  | reverse | GACCTGCCTGGGATCGGCAC |
| CXCL10 | forward | CCACGTGTTGAGATCATTGCCACG |
|  | reverse | ATCCATCGCAGCACCGGGGT |
| CXCL2 | forward | AGAAGTCATAGCCACTCTCAAGGGC |
|  | reverse | AGCAGCCCAGGCTCCTCCTTT |
| HPRT | forward | GTTAAGCAGTACAGCCCCAAA |
|  | reverse | AGGGCATATCCAACAACAAACTT |
| IL1α | forward | TCACCTTCAAGGAGAGCCG |
|  | reverse | ATCTGGGTTGGATGGTCTCTT |
| IL2 | forward | TGAGCAGGATGGAGAATTACAGG |
|  | reverse | GTCCAAGTTCATCTTCTAGGCAC |
| IL4 | forward | GAGCCATATCCACGGATGCGAC |
|  | reverse | ATGCGAAGCACCTTGGAAGCCC |
| IL5 | forward | CAGCTGTCCGCTCACCGAGCT |
|  | reverse | TTTCCACAGTACCCCCACGGACAG |
| IL6 | forward | ACAAAGCCAGAGTCCTTCAGAGAGA |
|  | reverse | AGCCACTCCTTCTGTGACTCCAG |
| IL10 | forward | GGCGCTGTCATCGATTTCTCCCC |
|  | reverse | GGCCTTGTAGACACCTTGGTCTTGG |
| IL12a | forward | CTGTGCCTTGGTAGCATCTATG |
|  | reverse | GCAGAGTCTCGCCATTATGATTC |
| IL12b | forward | AGTGTGAAGCACCAAATTACTCC |
|  | reverse | CCCGAGAGTCAGGGGAACT |
| IL13 | forward | TGCTTGCCTTGGTGGTCTCGC |
|  | reverse | GCGGCCAGGTCCACACTCCA |
| IL17a | forward | ACGCGCAAACATGAGTCCAGGG |
|  | reverse | TGAGGGATGATCGCTGCTGCCT |
| IL18 | forward | GTGAACCCCAGACCAGACTG |
|  | reverse | CCTGGAACACGTTTCTGAAAGA |
| IL23a | forward | GAACGCACATGCACCAGCGG |
|  | reverse | TGCAAGCAGAACTGGCTGTTGTCC |
| TNFα | forward | ACTTCGGGGTGATCGGTCCCC |
|  | reverse | TGGTTTGCTACGACGTGGGCTAC |
| TSLP | forward | ACGGATGGGGCTAACTTACAA |
|  | reverse | AGTCCTCGATTTGCTCGAACT |
| RXRα | forward | GATATCAAGCCGCCACTAGG |
|  | reverse | TGTTGTCTCGGCAGGTGTAG |
| RXRβ | forward | CACCTCTTACCCCTTCAGCA |
|  | reverse | GAGCGACACTGTGGAGTTGA |
| Notch1 | forward | TCAATGCCGTGGATGACCTA |
|  | reverse | CCTTGTTGGCTCCGTTCTTC |
| Notch2 | forward | GAGAAAAACCGCTGTCAGAATGG |
|  | reverse | GGTGGAGTATTGGCAGTCCTC |
| Rbpj | forward | AGTTGCACAGAAGTCTTACGG |
|  | reverse | CCTATTCCAATAAACGCACAGGG |
| TSLP-ChIP  proximal | forward | ATCTTAACCCAACCCACCAT |
|  | reverse | CTAGGGGAGGAACAGCTTCT |
| TSLP-ChIP  distal | forward | CCGTAGGCGTTTAGGTGTTA |
|  | reverse | CAAAGACTGTGCTCGGGTAT |
| TSLP-ChIP  3’-UTR | forward | TATTGCAAATCCAGCTGTCA |
|  | reverse | TTTCCAAAAGTGCTCACAAAA |
